# Can We Improve the Technique of Pelvic Floor Muscle Exercises in Postmenopausal Women Using a Single Electromyography Biofeedback Session? An Experimental Study

**DOI:** 10.3390/jcm13113062

**Published:** 2024-05-23

**Authors:** Magdalena Piernicka, Zbigniew Ossowski, Jakub Kortas, Damian Bojar, Justyna Labun, Anna Szumilewicz

**Affiliations:** Faculty of Physical Culture, Gdansk University of Physical Education and Sport, 80-336 Gdansk, Poland; zbigniew.ossowski@awf.gda.pl (Z.O.); jakub.kortas@awf.gda.pl (J.K.); bojar.damian.db@gmail.com (D.B.); justyna.labun@awf.gda.pl (J.L.); anna.szumilewicz@awf.gda.pl (A.S.)

**Keywords:** EMG, technique, Kegel muscles, quality of life, postmenopausal women

## Abstract

**Background**: The aim of this study was to assess the effect of a single session of EMG biofeedback in a group of postmenopausal women on improving technique in pelvic floor muscle (PFM) contractions (exercises). **Methods**: Sixty-two women aged 60 to 85 years (69 ± 4; mean ± SD) participated in the study. We assessed the technique of PFM exercises via surface electromyography (EMG) using a vaginal probe. A single assessment sequence consisted of 11 exercises involving the conscious contraction of the PFM, during which the order of activation for selected muscles was determined. We then awarded scores for exercise technique on a scale from 0 to 4, where 4 represented the best technique and 0 represented no activation of PFMs. In the second assessment, we used a biofeedback method to teach PFM exercise technique. **Results**: In total, 32% (n = 20) of the participants were unable to correctly perform the first PFM contraction, scoring 0.9 ± 0.79. After a single EMG biofeedback session, these women received 1.7 ± 1.08 scores (*p* = 0.003). In the tenth exercise, there was also a statistically significant improvement between the first (baseline) and second assessment (1.7 ± 1.34 and 2.15 ± 1.09, respectively; *p* = 0.037). For the remaining exercises, the results were not statistically significant, but we observed a positive trend of change. **Conclusions**: The use of a single EMG biofeedback session is an effective method of improving technique in PFM exercises in a group of women who initially performed them incorrectly.

## 1. Introduction

The pelvic floor muscles (PFMs) are a group of muscles consisting of three layers, extending from the symphysis pubis in front to the coccyx in the back, with the levator ani being the largest muscle. The functional importance of the PFMs involves two main roles. The first one is the support function for the pelvic organs, preventing organ prolapse. The second important function is participation in the mechanism of urinary continence, when the load on these muscles increases while they are performing various activities. The ability to relax the PFMs is essential inter alia during micturition [1,2].

As women age, the risk of PFM health problems, including urinary tract dysfunction, increases [3]. Urinary incontinence affects between 9% and 69% of postmenopausal women [4]. Menopause is defined as the period in a woman’s life after the cessation of menstruation, resulting from biological, genetic, and psychological factors. The state of menopause is associated with the loss of reproductive function, which most often occurs between the ages of 45 and 55 [5,6]. Symptoms caused by a decrease in estrogen levels cause many changes that affect the physical and mental health and quality of life of women [3,7]. According to the International Continence Society (ICS), uncontrolled leakage of urine through the urethra is classified as urinary incontinence [1]. Dysfunctions of the urinary system related to urinary incontinence are considered health, social, and hygiene problems affecting quality of life [1,8]. Based on the symptoms of leakage episodes and their pathophysiology, one can define the following types of urinary incontinence: stress urinary incontinence (SUI) occurring during physical activity, sneezing, coughing, lifting weight [1,9]; urgency incontinence (feeling of a sudden need to urinate and being unable to hold on until reaching the toilet); and mixed urinary incontinence (a combination of symptoms of stress and urgency incontinence) [9,10]. According to the recommendations of the International Continence Society, people with urinary incontinence symptoms are first recommended pelvic floor muscle training [11], which is effective in both the prevention [11,12,13] and treatment of urinary incontinence [11,12,14,15,16]. In addition, dysfunctions of the pelvic floor muscles have an influence on the deterioration of women’s sexual satisfaction [2,17].

Pelvic floor muscle training is defined as exercises aimed at improving strength, power, endurance, and the ability to relax in the pelvic floor muscles [1]. The effects of training reduce the symptoms of urinary incontinence, and this has a large impact on improving quality of life and self-esteem in women [15,16]. In order to increase the effectiveness of training, it is necessary to use the correct technique when performing exercises and motor control, enabling the conscious allocation of muscle contraction [1,18]. An inability to isolate the pelvic floor muscles during exercises may interfere with the training process and the expected results [19]. An example is co-contraction, which can act synergistically by increasing the activity of a selected muscle group or antagonistically, resulting in reduced activity in the pelvic floor muscles or replaced by the work of another muscle group (e.g., rectus abdominis muscles) [1]. A common problem, especially among people starting the training of pelvic floor muscles, is their correct location [20]. One of the methods of visualizing the neuromuscular activity of the pelvic floor muscles is the use of EMG biofeedback, referred to as feedback, which provides information on the activity performed [21]. The recorded muscle activity is displayed on the monitor, which effectively facilitates learning the correct exercise technique and remembering movement patterns [22,23]. Feedback and confidence in the correct performance of the PFM exercise is an important element for many women and may influence the motivation to continue the training process. In our previous studies conducted in a group of pregnant women, we confirmed the positive effect of a single biofeedback session on the firing order in which the pelvic floor muscles are activated and the contraction technique [24,25]. The aim of this study was to assess the effect of a single session of EMG biofeedback in a group of postmenopausal women on improving the technique of performing pelvic floor muscle contractions. In the second stage of the analyses, we aimed to verify the use of a single EMG biofeedback session, taking into account participants’ initial technique in performing pelvic floor muscle exercises, their age, and symptoms of urinary incontinence.

## 2. Materials and Methods

### 2.1. Participants

Sixty-two women aged 60 to 85 (69 ± 5; mean ± SD) participated in the study. We carried out the participant recruitment at the Universities of the Third Age in Gdansk and Sopot. Additional information about the project was posted on information boards in selected medical facilities and made available on social media. The eligibility criteria for the study included being at least 60 years of age and giving conscious consent to participate in the study. The criterion for exclusion from the study was a lack of consent from the healthcare physician to participate in the study.

We carried out all assessments at the Laboratory of Physical Effort and Genetics in Sport at Gdansk University of Physical Education and Sport (GUPES) in Poland. We conducted all research interventions in accordance with the principles of the WMA Helsinki Declaration and with the consent of Bioethics Commission at the Regional Medical Chamber in Gdansk (KB–5/22). Study participants signed informed-consent forms prior to testing. The research was carried out as part of the research project entitled, ‘Physical activity and selected indicators of the risk of disability in elderly people.’

### 2.2. Assessment of Neuromuscular Activity in Pelvic Floor Muscles and Implementation of a Biofeedback Session

We performed the evaluation of the technique of PFM contractions with the use of surface electromyography (EMG) with a vaginal electrode self-applied in the toilet by the participant. We used the Lifecare PR-02 Vaginal Electrode, Everyway Medical Instruments Co., Ltd., New Taipei City, Taiwan. Due to its streamlined shape, the electrode is comfortable to apply. Elements of the technical specification of the vaginal electrode are as follows: weight, 23.1 g; overall length, 76 mm; and diameter, 28 mm [26]. In order to control the technique for performing exercises, we glued surface electrodes to the synergistic muscles (rectus abdominis muscles, external oblique muscles, and gluteus maximus). We used disc electrodes (SKINTACT Premier W-60, LEONHARD LANG GmbH, Innsbruck, Austria) [26].

Before the assessment, we asked the participants to assume the position of lying on their back, legs bent at the knees and arms along the body. The women received information on how to contract their PFMs correctly, leaving the synergistic muscles relaxed. In order to better clarify the task, we instructed the participants to perform a PFM contraction as if they wanted to stop the stream of urine.

We conducted the assessments of the neuromuscular activity of the PFMs based on the Glazer protocol [27]. Participants performed two consecutive sequences of PFM contractions, the first blinded and the second with the EMG biofeedback method. A single sequence consisted of eleven individual exercises of various lengths of contraction. According to recommendations, the participant received information about performing maximum contractions of the PFMs [28]. During the second sequence of exercises, participants watched changes in PFM activity on a computer screen (with biofeedback). The method used aimed to increase awareness of the contractions performed, by learning how to locate and isolate the PFMs and remembering the correct movement patterns [23].

To assess technique in PFM exercises, we used the MyoResearch program (XP Master Edition 1.08.32), which determines the activation of selected muscles and their order (so called ‘firing order’). Activity of a given muscle was recorded when the EMG value exceeded three times the standard deviation of the patient’s baseline value. In order to obtain reliable data, a minimum duration of 0.2 s of muscle activity in contraction above the EMG threshold value was determined, thus avoiding noting single-spike disturbances [29]. We carried out all assessments under the same conditions for all participants by using a program with automatic verbal instructions. Automatic contraction and relaxation commands enabled one standard to be maintained for exercise performance.

### 2.3. Assessment of Pelvic Floor Muscle Exercise Techniques

Based on the EMG records, we assessed the firing order of muscles involved in individual exercises on a score scale ranging from 4 to 0 [30]. The highest-rated exercise technique was the activity of PFMs while maintaining the relaxation of the synergistic muscles. We assigned a score of three to the exercise technique performed with the activation of PFMs before the synergistic muscles. We assigned a score of two to a technique in which the synergistic muscles were activated first and the PFMs were activated later. We gave a score of one when we observed a lack of activity in the PFMs and the contraction of other muscles. Zero scores were assigned in the absence of any muscle activity in a given exercise (i.e., neither PFM nor synergistic muscle activity was recorded).

Participants were classified for further analysis based on the technique of the first exercise performed. Correct technique group (n = 42) included women who received scores of 3 and 4 for the technique in the first exercise. Women who were scored 0–2 in the first exercise were assigned to the incorrect technique group. In the later part of the analysis, we analyzed the effect of a single biofeedback session on the improvement of technique among women who performed the first exercise with PFM activation after synergistic muscles or did not activate the PFMs at all.

### 2.4. Assessment of the Impact of Urinary Incontinence on Quality of Life

All participants self-completed Incontinence Impact Questionnaires (IIQs), enabling the assessment of the impact of urinary incontinence symptoms on their quality of life. IIQs are used as reliable tools to detect various urogenital symptoms and their degree of severity. The use of an ordinal scale allows the identification of participants who experienced an impact from urinary incontinence symptoms on their quality of life [31,32]. Dividing the participants according to the reported impact of urinary incontinence (varying degrees of severity) allowed further analyses to verify whether a single EMG biofeedback session was important in improving the technique of pelvic floor muscle exercises in the symptomatic and asymptomatic groups.

### 2.5. Description of Statistics

We performed statistical analysis using Statistica 13.1 software. All values are expressed as mean ± standard deviation (SD). We applied the Shapiro–Wilk test to assess the homogeneity of dispersion from the normal distribution. We analyzed the differences between the baseline and second assessments using the Wilcoxon rank test. Statistical significance was set at *p* < 0.05. We determined the sample size by using a power calculation with the software G∗power version 3.1.9.4. The analysis of the sample size showed that a sample of 54 participants was sufficient to achieve the assumed level of statistical significance (α = 0.05, β = 0.95, d = 0.5).

## 3. Results

In Table 1, we present the characteristics of the study participants, taking into account variables that may affect the functioning of the pelvic floor muscles and symptoms of urinary incontinence: age, BMI, and parity (Table 1). We did not note any statistically significant differences between the subgroups classified according to the technique of exercising the pelvic floor muscles in the first exercise. Older participants and those who reported symptoms of urinary incontinence had a statistically significantly higher BMI. Additionally, the symptomatic group reported statistically more deliveries. Our outcomes are consistent with the reports of other researchers [1,4].

In Table 2, we present the scores obtained for technique in exercises performed for the baseline assessment and with the use of the biofeedback method (Table 2). The use of a single biofeedback session among all participants did not show statistically significant changes. However, we observed a positive trend in most exercises.

**Figure 1:**
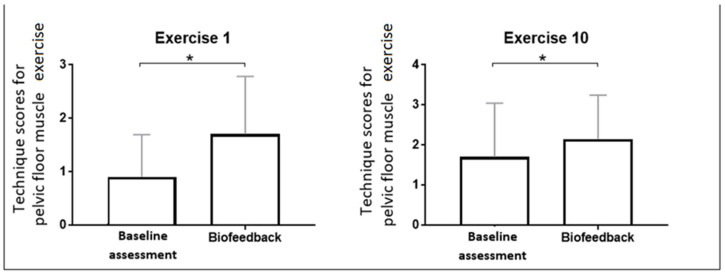
Changes in the technique scores for the first and tenth pelvic floor muscle exercises after a single EMG biofeedback session in the subgroup presenting incorrect technique at baseline; * was considered statistically significant.

We carried out further analysis of the results to assess the effectiveness of a single EMG biofeedback session in women initially performing PFM exercises with incorrect technique (Table 3). We selected a group of women who showed incorrect technique in PFM contractions in the first exercise (receiving scores from 0 to 2 for technique in the first exercise). Worryingly, one in three women in the postmenopausal study group was unable to perform the first PFM exercise correctly, scoring 0.9 ± 0.79. After the biofeedback session, these women achieved 1.7 ± 1.08 scores for technique (*p* < 0.001) (Figure 1). In the tenth exercise, there was also a statistically significant improvement between the baseline and second assessment (1.7 ± 1.34 and 2.15 ± 1.09, respectively; *p* = 0.037) (Figure 1). In the remaining exercises, the results were not statistically significant, but a positive trend of change was observed.

Another variable in our analysis to verify the influence of a single EMG biofeedback session on technique improvement was the age group of women in the study (Table 4). We assigned the participants aged 60–69 (n = 30; 65 ± 2) to the first group, while women over 70 (n = 32; 73 ± 3) were assigned to the second group. The obtained results showed no statistical significance in terms of improving exercise technique between age groups. In both groups, we observed a slight improvement in technique in individual PFM exercises in the assessment using the EMG biofeedback method.

We also tested the use of a single biofeedback session in relation to urinary incontinence symptoms (Table 5). For this purpose, we divided the study participants into two groups based on the results of the IIQ questionnaire. The first group included women who did not report any symptoms of urinary incontinence (n = 28). Participants who reported any impact of urinary incontinence on their quality of life were assigned to the second group (n = 34). The analysis that took into account the symptoms of urinary incontinence did not show statistically significant changes between the symptomatic and asymptomatic groups, either.

## 4. Discussion

The most important finding from our study is that a single EMG biofeedback session is effective in improving PFM exercise technique in a group of women who presented incorrect exercise technique in the first exercise at baseline. We observed statistically significant changes in technique in the first and tenth exercises in the sequence of eleven exercises. We did not observe any negative changes in the remaining exercises. On the contrary, a positive trend was recorded in most of the observations. The obtained results encourage further research, in particular, to verify the impact of a larger number of EMG biofeedback sessions on improving technique in PFM exercises in postmenopausal women.

To respond to the aim of the study, we assessed technique in PFM exercises using EMG in a blind assessment and after a single EMG biofeedback session. The disturbing result is that one in three postmenopausal women could not properly perform PFM exercises. This outcome indicates a need for greater popularization of PFM exercises in women of this age group. In order to increase the effectiveness of PFM training and improve pelvic floor function, attention should be paid to the correct exercise technique [33]. Some researchers noted that simultaneous contractions of synergistic muscles may disturb the awareness and strength of PFM contractions [34]. The most common errors in technique in PFM exercises include contractions of synergistic muscles and performing pelvic movements in addition to, or instead of, PFM contractions [19,35]. By using visual and verbal feedback during exercises, one can effectively unlearn errors in the exercise technique [35]. The use of EMG is an excellent method for assessing the function of the PFMs [36,37]. By processing the EMG signal, we were able to precisely determine the sequence of activation of selected muscles, which we translated into an assessment of contraction technique in the individual exercises [38].

In 2023, Lopéz-Pérez et al. published a literature review on PFM exercises in the treatment of urinary incontinence in postmenopausal women. All the analyzed publications (n = 8) reported scientific evidence supporting the use of PFM training as an effective intervention in the treatment of urinary incontinence in the study population. However, none of the authors used the EMG biofeedback method. Six of the eight studies also used a questionnaire to assess the impact of urinary incontinence on quality of life [39]. The available literature lacks studies assessing the impact of a single EMG biofeedback session on improving PFM exercise technique in postmenopausal women. In our previous research, we analyzed the impact of a single EMG biofeedback session in a group of pregnant women, both in terms of the exercise technique and the level of neuromuscular activity of the pelvic floor muscles [24,25]. Using the existing knowledge regarding the positive impact of EMG biofeedback on learning to locate and isolate the PFMs and consolidation of correct movement patterns and improving the effectiveness of training, we decided to transfer it to a group of postmenopausal women.

We did not observe differences in improving the technique of PFM exercises using a single EMG biofeedback session between symptomatic and asymptomatic groups, classified based on IIQ. This result may be due to the too-small group of participants, which made it impossible to divide the group according to the type and severity of urinary incontinence. In the future, it would be worth verifying this research issue in a larger number of respondents, considering each aspect of the impact of urinary incontinence symptoms on quality of life. Nevertheless, a surprising result was that women who experienced some symptoms of urinary incontinence did not show significantly worse technique in pelvic floor muscle exercises. In further research, it would be worth conducting this type of study, taking into account the long-term effectiveness of the intervention, which would significantly increase the value of the experiment.

In a study assessing the effectiveness of PFM training with and without EMG biofeedback in the treatment of urinary incontinence, Hagen et al. examined a total of 593 women. Their study did not show statistically significant differences in the severity of urinary incontinence symptoms after 24 months between the group receiving PFMT using the EMG biofeedback method and the group receiving only PFMT [23]. In our research, statistically significant changes were observed in the improvement of exercise technique in women who demonstrated incorrect exercise technique in the first exercise. Among the study participants who performed the exercise with the correct technique, there could be no improvement in the scores obtained for the technique because they received the maximum score (4) in the baseline assessment. According to the recommendations of the National Institute for Health and Care Excellence [40], the use of the EMG biofeedback method should be recommended for learning proper exercise techniques, and not as part of routine training. Our results were consistent with the above recommendations. The use of the EMG biofeedback method in each training involves additional costs; therefore, it is important to note that the use of this method seems to be necessary and effective for people starting a training program. The number of EMG sessions could be increased for participants who do not achieve the expected results in terms of learning PFM exercise technique after a single EMG biofeedback session.

In this study, the factor determining the correctness of the first exercise was not taken into account when calculating the sample size. Taking this variable into account could reveal more statistically significant changes, as this subgroup had a small number of people in this study. It is worth conducting further research in which the correctness or incorrectness of the first pelvic floor muscle exercise will constitute the inclusion criterion. The lack of a parallel control group in the assessment of the effectiveness of a single EMG biofeedback session in a group of postmenopausal women is another limitation of our study.

## 5. Conclusions

The use of a single EMG biofeedback session is an effective method for improving technique in PFM exercises in a group of women who performed the exercises incorrectly at baseline. Considering the importance of proper PFM exercise technique for the effects of training programs, the EMG biofeedback method should be widely used to teach the correct location and isolation of this muscle group. In the study participants who performed the exercise with the correct technique, there could be no improvement in the scores obtained for the technique because they received the maximum score (4) in the baseline assessment. In addition, we observed that neither age nor urinary incontinence symptoms were factors determining exercise technique in our study group.

## 6. Perspective

Our aim in developing our research is to verify whether multiple repetitions of the EMG biofeedback session would further improve the technique of pelvic floor muscle exercises in postmenopausal women. In the long term, it would be worth conducting the study in a group of postmenopausal women diagnosed with severe urinary incontinence. Another interesting issue to assess is technique in pelvic floor muscle exercises in a group of women with other dysfunctions, including sexual dysfunctions, pelvic organ prolapse, and fecal incontinence.

## Figures and Tables

**Table 1:** Characteristics of the study participants.

Variables	All Participants (n = 62)		
**Age**	69.23 ± 4.83		
**BMI (kg∙m^−2^)**	27.37 ± 4.01		
**Parity**	1.60 ± 0.79		
**IIQ score**	17.84 ± 21.43		
	**Incorrect technique** **(n = 20)**	**Correct technique** **(n = 42)**	***p*-value**
**Age**	70.25 ± 3.31	68.74 ± 5.37	0.252
**BMI (kg∙m^−2^)**	28.39 ± 3.55	26.88 ± 4.16	0.168
**Parity**	2.00 ± 1.12	1.93 ± 1.05	0.997
**IIQ score**	22.12 ± 26.92	15.70 ±18.1	0.603
	**Age 60–69 (n = 30)**	**Age > 70 (n = 32)**	***p*-value**
**BMI (kg∙m^−2^)**	26.07 ± 4.07	28.59 ± 3.59	0.016 *
**Parity**	1.92 ± 0.86	2 ± 1.24	0.882
**IIQ score**	13.12 ± 14.64	22.25 ± 25.72	0.284
	**Asymptomatic group** **(IIQ = 0); (n = 28)**	**Symptomatic Group** **(IIQ > 0); (n = 34)**	***p*-value**
**Age**	69 ± 5.63	69.36 ± 4.13	0.552
**BMI (kg∙m^−2^)**	26.2 ± 3.5	28.26 ± 4.3	0.049 *
**Parity**	1.44 ± 0.75	2.36 ± 1.11	0.003 *

Values are means ± SD. BMI—body mass index, IIQ—Incontinence Impact Questionnaire, ***p* < 0.05** was considered statistically significant *.

**Table 2:** Changes in the technique scores for pelvic floor muscle exercises after a single EMG biofeedback session in all study participants.

	All Participants (n = 62)		
Exercise Number	Baseline Assessment	Biofeedback	*p*-Value
Exercise 1	2.68 ± 1.38	2.61 ± 1.23	0.616
Exercise 2	2.47 ± 1.57	2.63 ± 1.36	0.36
Exercise 3	2.4 ± 1.55	2.65 ± 1.4	0.169
Exercise 4	2.47 ± 1.61	2.53 ± 1.48	0.668
Exercise 5	2.6 ± 1.45	2.71 ± 1.37	0.568
Exercise 6	2.56 ± 1.07	2.61 ± 0.98	0.657
Exercise 7	2.65 ± 1.24	2.73 ± 0.99	0.681
Exercise 8	2.6 ± 1.19	2.66 ± 1.02	0.781
Exercise 9	2.58 ± 1.15	2.52 ± 1.08	0.496
Exercise 10	2.66 ± 1.17	2.63 ± 0.89	0.792
Exercise 11	2.52 ± 0.82	2.61 ± 0.8	0.411

Values are presented as mean ± standard deviation (SD); *p* < 0.05 was considered statistically significant; the differences were assessed with the Wilcoxon rank test; the 0–4 scale for the exercise technique takes into account the isolation of PFMs from synergistic muscles: 4—best technique; 0—incorrect technique (details on the exercise technique scores are presented in the Section 2.3).

**Table 3:** Changes in the technique scores for pelvic floor muscle exercise after a single EMG biofeedback session in the subgroup presenting incorrect technique in the first exercise at baseline.

Women Presenting Incorrect Technique (n = 20) in the First Exercise at Baseline			
Exercise Number	Baseline Assessment	Biofeedback	*p*-Value
Exercise 1	0.9 ± 0.79	1.7 ± 1.08	0.003 *
Exercise 2	1 ± 1.12	1.5 ± 1.28	0.948
Exercise 3	1.05 ± 1.28	1.6 ± 1.47	0.605
Exercise 4	1.05 ± 1.15	1.5 ± 1.32	0.709
Exercise 5	1.6 ± 1.43	1.65 ± 1.27	0.575
Exercise 6	1.8 ± 1.06	1.8 ± 1.15	0.67
Exercise 7	1.55 ± 1.19	2.1 ± 1.37	0.334
Exercise 8	1.6 ± 1.14	2.15 ± 1.04	0.207
Exercise 9	1.55 ± 1.19	1.9 ± 1.17	0.064
Exercise 10	1.7 ± 1.34	2.15 ± 1.09	0.037 *
Exercise 11	1.9 ± 0.79	2.15 ± 1.14	0.814

Values are presented as mean ± standard deviation (SD); ***p* < 0.05** was considered statistically significant *; the differences were assessed with the Wilcoxon rank test; the 0–4 scale for exercise technique takes into account the isolation of PFMs from synergistic muscles: 4—best technique; 0—incorrect technique (details on the exercise technique scores are presented in the Section 2).

**Table 4:** Changes in the technique scores for pelvic floor muscle exercises after a single EMG biofeedback session in two age-based subgroups of study participants.

Exercise Number	Age 60–69 (n = 30)			Age > 70 (n = 32)		
	Baseline Assessment	Biofeedback	*p*-Value	Baseline Assessment	Biofeedback	*p*-Value
Exercise 1	2.93 ± 1.23	2.83 ± 1.05	0.496	2.44 ± 1.48	2.41 ± 1.36	0.888
Exercise 2	2.53 ± 1.59	2.77 ± 1.3	0.394	2.41 ± 1.56	2.5 ± 1.41	0.807
Exercise 3	2.57 ± 1.5	2.8 ± 1.27	0.333	2.25 ± 1.61	2.5 ± 1.52	0.32
Exercise 4	2.63 ± 1.59	2.7 ± 1.26	0.836	2.31 ± 1.64	2.38 ± 1.66	0.704
Exercise 5	2.67 ± 1.4	2.93 ± 1.11	0.345	2.53 ± 1.52	2.5 ± 1.57	0.968
Exercise 6	2.67 ± 0.99	2.5 ± 1.07	0.397	2.47 ± 1.14	2.72 ± 0.89	0.187
Exercise 7	2.6 ± 1.3	2.73 ± 0.83	0.594	2.69 ± 1.2	2.72 ± 1.14	0.979
Exercise 8	2.73 ± 1.14	2.6 ± 0.97	0.46	2.47 ± 1.24	2.72 ± 1.08	0.334
Exercise 9	2.63 ± 1.19	2.43 ± 1.1	0.301	2.53 ± 1.14	2.59 ± 1.07	0.807
Exercise 10	2.67 ± 1.27	2.5 ± 0.97	0.352	2.66 ± 1.1	2.75 ± 0.8	0.529
Exercise 11	2.63 ± 0.76	2.7 ± 0.53	0.575	2.41 ± 0.87	2.53 ± 0.98	0.552

Values are presented as mean ± standard deviation (SD); *p* < 0.05 was considered statistically significant; the differences were assessed with the Wilcoxon rank test; the 0–4 scale for the exercise technique takes into account the isolation of PFMs from synergistic muscles: 4—best technique; 0—incorrect technique (details on the exercise technique scores are presented in the Section 2.3).

**Table 5:** Changes in the technique scores for pelvic floor muscle exercises after a single EMG biofeedback session in subgroups of asymptomatic and symptomatic study participants in relation to the impact of urinary incontinence on their quality of life.

Exercise Number	Asymptomatic Group (IIQ = 0); (n = 28)			Symptomatic group (IIQ > 0); (n = 34)		
	Baseline Assessment	Biofeedback	*p*-Value	Baseline Assessment	Biofeedback	*p*-Value
Exercise 1	2.74 ± 1.38	2.78 ± 1.05	0.859	2.61 ± 1.43	2.52 ± 1.37	0.737
Exercise 2	2.41 ± 1.55	2.56 ± 1.45	0.776	2.52 ± 1.64	2.67 ± 1.34	0.433
Exercise 3	2.48 ± 1.53	2.59 ± 1.47	0.722	2.39 ± 1.58	2.67 ± 1.41	0.248
Exercise 4	2.22 ± 1.74	2.52 ± 1.58	0.272	2.7 ± 1.51	2.52 ± 1.46	0.614
Exercise 5	2.41 ± 1.5	2.59 ± 1.5	0.754	2.79 ± 1.43	2.76 ± 1.3	0.996
Exercise 6	2.59 ± 1.12	2.63 ± 0.97	0.824	2.55 ± 1.06	2.61 ± 1.03	0.629
Exercise 7	2.56 ± 1.25	2.78 ± 0.89	0.48	2.76 ± 1.25	2.7 ± 1.1	0.717
Exercise 8	2.59 ± 1.22	2.81 ± 0.74	0.3	2.58 ± 1.23	2.64 ± 1.14	0.974
Exercise 9	2.56 ± 1.19	2.33 ± 1.27	0.262	2.58 ± 1.17	2.7 ± 0.88	0.695
Exercise 10	2.56 ± 1.28	2.7 ± 0.82	0.507	2.73 ± 1.13	2.58 ± 0.97	0.293
Exercise 11	2.56 ± 0.8	2.52 ± 0.85	0.859	2.48 ± 0.87	2.7 ± 0.77	0.158

Values are presented as mean ± standard deviation (SD); *p* < 0.05 was considered statistically significant; the differences were assessed with the Wilcoxon rank test; the 0–4 scale for the exercise technique takes into account the isolation of PFMs from synergistic muscles: 4—best technique; 0—incorrect technique (details on the exercise technique scores are presented in the Section 2.3). IIQ—Incontinence Impact Questionnaire; asymptomatic group—participants who scored zero points in the IIQ questionnaire; symptomatic group—participants who reported any symptoms of urinary incontinence having an impact on quality of life in the IIQ questionnaire.

## Data Availability

Due to privacy legislation, we are unavailable to provide the full data.
